# A Study on Highly Effective Electromagnetic Wave Shield Textile Shell Fabrics Made of Point Polyester/Metallic Core-Spun Yarns

**DOI:** 10.3390/polym14132536

**Published:** 2022-06-21

**Authors:** Chen-Hung Huang, Po-Wen Hsu, Zhao-We Ke, Jian-Hong Lin, Bing-Chiuan Shiu, Ching-Wen Lou, Jia-Horng Lin

**Affiliations:** 1 Department of Aerospace and Systems Engineering, Feng Chia University, Taichung 40724, Taiwan; chhuang@fcu.edu.tw; 2 College of Material and Chemical Engineering, Minjiang University, Fuzhou 350108, China; j0928451554@gmail.com; 3 Advanced Medical Care and Protection Technology Research Center, Department of Fiber and Composite Materials, Feng Chia University, Taichung 07102, Taiwan; ss343300@gmail.com (Z.-W.K.); baron.lin69@gmail.com (J.-H.L.); 4 Laboratory of Fiber Application and Manufacturing, Department of Fiber and Composite Materials, Feng Chia University, Taichung 40724, Taiwan; 5 Fujian Key Laboratory of Novel Functional Fibers and Materials, Minjiang University, Fuzhou 350108, China; 6 Department of Bioinformatics and Medical Engineering, Asia University, Taichung 413305, Taiwan; 7 Department of Medical Research, China Medical University Hospital, China Medical University, Taichung 404333, Taiwan; 8 Advanced Medical Care and Protection Technology Research Center, College of Textile and Clothing, Qingdao University, Qingdao 266071, China; 9 School of Chinese Medicine, China Medical University, Taichung 404333, Taiwan

**Keywords:** Ge, core-spun yarns, negative ion, electromagnetic interference shielding effectiveness (EMI SE), stainless steel filaments

## Abstract

In this study, stainless steel (SS) filaments are wrapped in Ge fibers to form core-spun yarns. The yarns along with 500 D polyester (PET) fibers undergo weaving, thereby forming functional woven fabrics. The experiment is composed of two parts:yarns and fabrics. The yarns are twisted with TPI of 8, 9, 10, 11, and 12, and then tested for tensile strength and tensile elongation. The yarns possess mechanical properties that are dependent on the TPI—the higher the TPI, the better the mechanical properties. The maximal mechanical properties occur when the core-spun yarns are made of 12 TPI where the maximal tensile strength is 5.26 N and the lowest elongation is 43.2%. As for the functional woven fabrics, they are made of Ge/SS core-spun yarns as the weft yarns and 500 D PET yarns as the warp yarns. The tensile strength, tensile elongation, negative ion release, electromagnetic interference shielding effectiveness (EMI SE), and air permeability tests are conducted, determining the optimal woven fabrics. The 12 TPI core-spun yarns provide the woven fabrics with the maximal tensile strength of 153.6 N and the optimal elongation at break of 10.08%. In addition, the woven fabrics made with 8 or 9 TPI core-spun yarns exhibit an optimal EMI SE of 41 dB, an optimal air permeability of 212 cm^3^/cm^2^/s, and an optimal release amount of negative ion of 550–600 ions/cc. The proposed woven fabrics have a broad range of applications, such as functional garments and bedding.

## 1. Introduction

The benefit of advanced technology in a modern society is to allow people to have a convenient life supported by electronic instrumentation. However, people show a growing tendency depending on the electronic instrumentation, which subsequently inflicts them with a decrease in immunity, changes in metabolism, and a rise in blood pressure [1,2,3,4,5,6]. The reduction of electromagnetic waves via shields thus becomes an important study. As for personal electromagnetic protective gear, the protective clothing makes a good research topic. There are numerous studies indicating that fabrics demonstrate good electromagnetic wave shielding efficacy when composed of stainless steel [7,8,9,10].

Electromagnetic interference shielding effectiveness (EMI SE) is a function that can block electromagnetic waves, involving complex and challenging techniques. Electromagnetic waves generate electromagnetic interference (EMI) that renders instruments or facilities with malfunction [11]. To protect people from harm caused by electromagnetic waves, many scholars have been exploring measures to reduce or prevent the negative influence of EMI [12,13,14,15,16,17,18]. Stainless steel wires are mixed with yarns to form woven fabrics, after which the electromagnetic wave shielding test is conducted, thereby proving that the plain-woven fabrics outperform the other patterns in terms of EMI SE [19,20,21,22,23]. In addition, the porosity and surface resistance are two crucial factors to the electromagnetic wave shielding performance [24], and fabrics made of finer fibers show the maximal EMI SE [25]. Besides, stainless steel/polyester conductive fabrics also have EMI protection, and the efficacy is highly associated with the weft yarn density, the weft yarn ratio, the conductive yarn ratio, the stainless-steel ratio, the fabric pattern, and the number of fabric laminates. A rise in the fabric-and-yarn lamination layers has a positive influence on the EMI of fabrics [26]. In particular, woven fabrics consisting of stainless steel wires show excellent EMI protection at high frequencies, achieving as high as 40 dB [27]. There are newly developed electromagnetic interference studies. The hot plasticity composites composed of carbon nanotubes and thermoplastic polyurethane (TPU) could attain 37.4 dB [28]. The chlorinated polyethylene composites composed of nanometer carbon fibers and carbon black could block electromagnetic interference of 22.5~25 dB and 22.5~25 dB with a thickness of 0.5 mm and 2 mm, respectively [29]. Moreover, cotton fabrics coated with carbon black presented electric conductivity and electromagnetic interference [30]. Electromagnetic interference materials composed of carbon-fiber woven fabric and effervescence could attain 45~60 dB with variations in stacking layers [31]. Similarly, electromagnetic interference composites composed of carbon-fiber woven fabric and graphene coating layer exhibited 18 dB [32]. In addition, electromagnetic interference composites made of carbonyl iron powder (CIP) and acrylonitrile butadiene styrene copolymers (ABS) via a 3D printer demonstrated a high degree of freedom, providing a flexible range of requirement by the electromagnetic shielding purposes [33].

The constantly developing urbanization also means that people spend most of their time living in a city, therefore enduring severe pollution, which causes diverse syndromes. A shortage of negative ions is also ascribed for poor health [34]. Negative ions can improve melancholy and enhance felicity, alleviating the allergy caused by dust, fungus spores, and allergens [35]. Germanium (Ge) elements release negative ions when at 32 °C, and can free people from the long-term exposure to positive-ions [36]. In this study, stainless steel filaments and Ge fibers are formed into core-spun yarns with a ring frame. The core-spun yarns are composed with twist per inch (TPI) of 8, 9, 10, 11, and 12, and are then tested for tensile strength and elongation, which helps determining the influence of the TPI. Next, the optimal core-spun yarns are made into woven fabrics that are subsequently tested for fabric tensile strength, fabric elongation, electromagnetic wave shielding, negative ion release, and air permeability. It is hoped that the proposed woven fabrics can yield advantages of both stainless steel (i.e., electromagnetic wave shielding) and Ge (i.e., negative ion release), and serve as the fabrics that are beneficial to the health of users.

## 2. Experimental

### 2.1. Materials

Ge fibers (Formosa Taffeta Co., Ltd., Taichung, Taiwan) are composed of 65% of cotton fibers and 35% of Ge fibers. Stainless steelwires (Yuen Neng Co., Ltd., Taichung, Taiwan) have a diameter of 0.065 mm. Polyethylene terephthalate (PET) filaments (Universal Textile Co., Ltd., Taichung, Taiwan) are 500 de-nier (D) and have a diameter of 0.065 mm.

### 2.2. Methods

**Figure 1:**
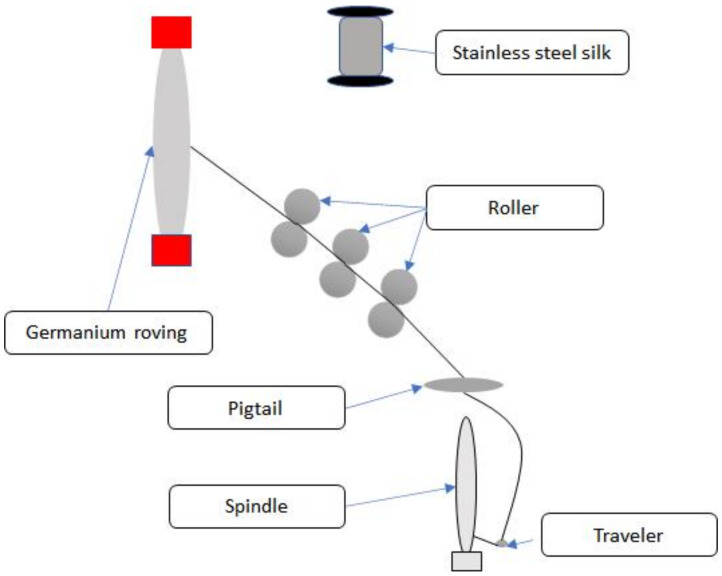
Diagram of a ring frame.

**Figure 2:**
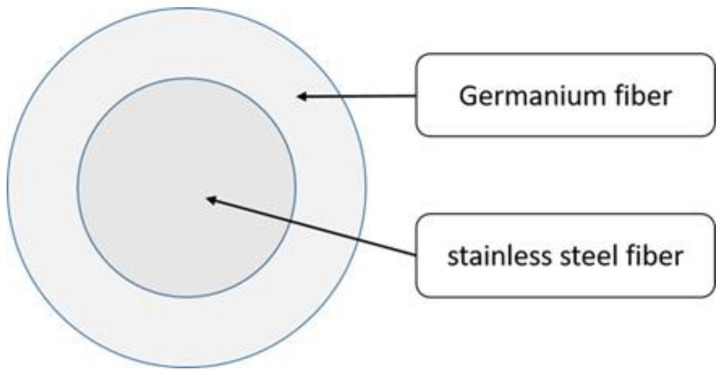
Diagram showing the composition of core-spun yarns.

Ge fibers are fed into a ring frame (SM-06, Sun Mien Mechanical Co., Ltd., Taichung, Taiwan) through a funnel entry, a rear rolla, and a middle rolla, during which stainless steel filaments are fed through a V-groove guide wheel and two rolla, and a ballooning eye, and then coiled over a spindle. Subsequently, the stainless-steel filaments are wrapped in Ge fibers, forming core-spun yarns that then pass a ballooning eye and steel clip and are eventually collected over a spindle. the specific process of which is shown in Figure 1. The TPI is computed with Equation (1), and Figure 2 shows the composition of the core-spun yarns.

(1) $$\mathrm{TPI}=10.641\times \frac{\mathrm{Number}\;\mathrm{of}\;\mathrm{teeth}\;\mathrm{on}\;\mathrm{upper}\;\mathrm{gear}}{\mathrm{Number}\;\mathrm{of}\;\mathrm{teeth}\;\mathrm{of}\;\mathrm{lower}\;\mathrm{gear}}.$$

**Figure 3:**
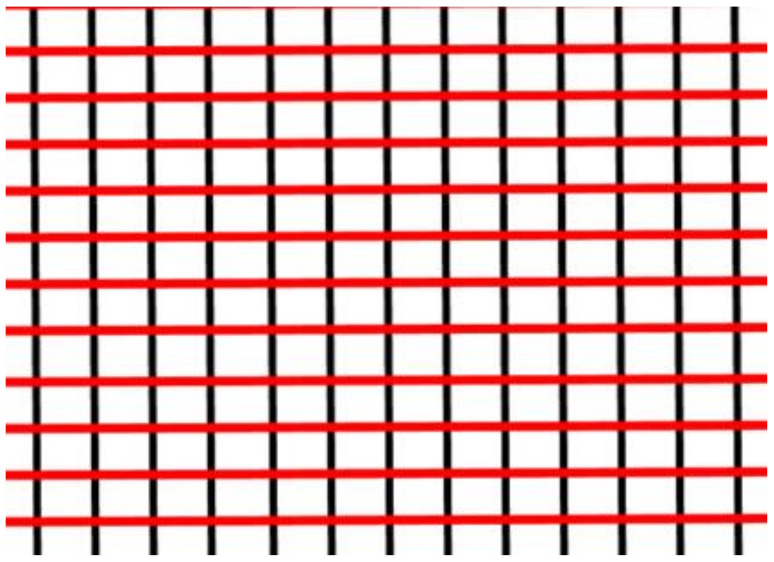
The woven fabrics are composed of weft yarns (illustrated in red) and warp yarns (illustrated in black).

The yarns are collected over a paper tube via an automatic yarn winder (Nan Hsing Machine Factory, Taichung, Taiwan), facilitating the subsequent tests and weaving. Core-spun yarns made with different TPI serve as the weft yarns while 500 D PET yarns serve as the warp yarns to form woven fabrics using a sword mast shuttle loom (KINGSTON280S, King Kon Iron Works, Ltd., Changhua, Taiwan). shows the configuration of woven fabrics the structure is shown in Figure 3.

### 2.3. Testing

#### 2.3.1. Tensile Performances of the Core-Spun Yarns

A universal tester (HT-2402, Hung Ta Instrument, Taichung, Taiwan) is used to measure the tensile performances of Ge/SS core-spun yarns at a test rate of 300 mm/min as specified in ASTM D 2256. The distance between clamps is 250 mm and 20 samples for each specification are tested for the average.

#### 2.3.2. Tensile Performances of the Non-Woven Fabrics

As specified in ASTM D 5035-11 (2015) (Strip Method), the tensile strength and elongation at the break of non-woven fabrics are characterized using a universal tester (Hung Ta Instrument, Taichung, Taiwan). The distance between clamps is 200 mm and the test rate is 300 mm/min. Ten samples are taken along the MD and CD for each specification.

#### 2.3.3. Electromagnetic Interference Shielding Effectiveness (EMI SE) of Non-Woven Fabrics

The electromagnetic wave shielding of non-woven fabrics is measured as specified in ASTM D4935-18. The electromagnetic wave shielding system applies test clamps (Electro-Metrics Corporation, Johnstown, NY, USA) that run at a frequency range of 1~3 GHz. A blank specimen with an identical thickness is measured for electromagnetic wave shielding as the control group (SERef) and used for rectification of the tester.

#### 2.3.4. Air Permeability of Non-Woven Fabrics

The air permeability of non-woven fabrics is measured using a tester (FX3300, TEXTEST, Schwerzenbach, Switzerland) as specified in ASTM D737-041. According to the standard, the air pressure is 125 Pa and samples have a size of 250 mm × 250 mm.

#### 2.3.5. Negative Ion Release of Non-Woven Fabrics

The negative ion release of woven fabrics is measured using a negative ion tester (ITC-201A, Andes Electric Co., Ltd., Hachinohe City, Japan). The indoor environment has a relative humidity 65 ± 5% RH and a temperature of 32 ± 2 °C. Samples have a size of 300 mm × 200 mm. The length of test time is 15 min. Five samples for each specification are used for the average.

## 3. Results and Discussion

### 3.1. Tensile Strength and Elongation of Ge/SS Core-Spun Yarns as Related to TPI

**Figure 4:**
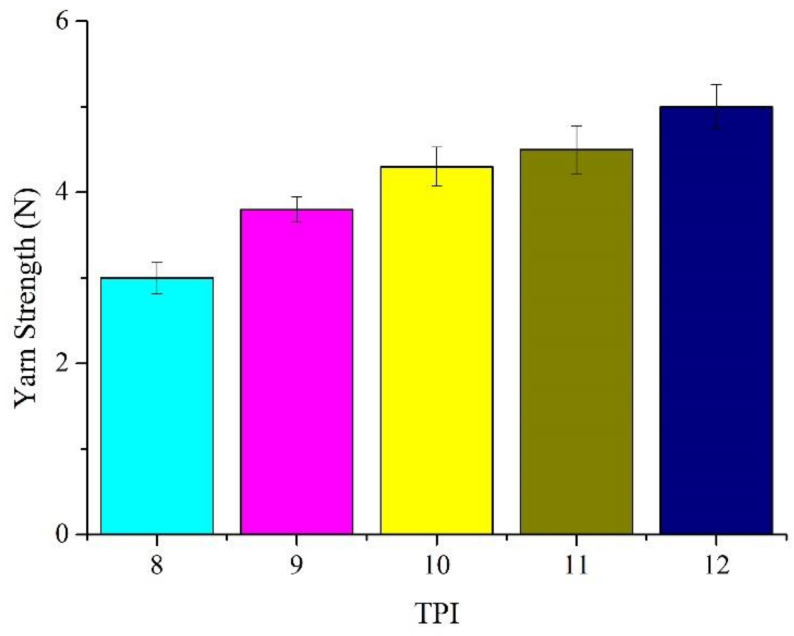
Tensile strength of the Ge/SS core-spun yarns.

Table 1 shows that the tensile strength of Ge/SS core-spun yarns is proportional to the TPI, which consistent with the findings of other studies [37]. A higher TPI enhances the fiber cohesion between the Ge staple fibers and stainless steel filaments [15]. Based on Figure 4, the tensile strength of the core-spun yarns increases as the TPI increases. From 8 to 12 TPI, the tensile strength is improved by 66%, reaching a maximal tensile strength of 5.0 N. Additionally, when TPI is increased to 12, the over-twisting is not presented. The CV% of each TPI is lower than 10%, which suggests that the yarns have good reproducibility.

**Figure 5:**
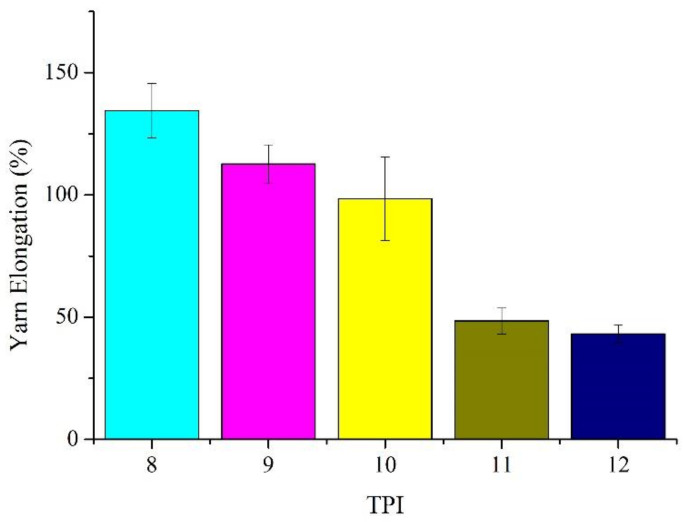
Tensile elongation of the Ge/SS core-spun yarns.

Table 1 and Figure 4 and Figure 5 show that the core-spun yarns have tensile elongation that is inversely proportional to the TPI. A higher TPI renders a twisting effect over the stainless-steel filaments, which compromises the ductility of stainless-steel filaments. A rise in the TPI exerts a more powerful twisting force over the stainless-steel filaments, reducing the tensile elongation to a greater extent. As a result, the tensile elongation decreases by 70% when TPI is increased from 8 to 12.

### 3.2. Tensile Strength and Elongation of Woven Fabrics as Related to TPI of Constituent Ge/SS Core-Spun Yarns

**Figure 6:**
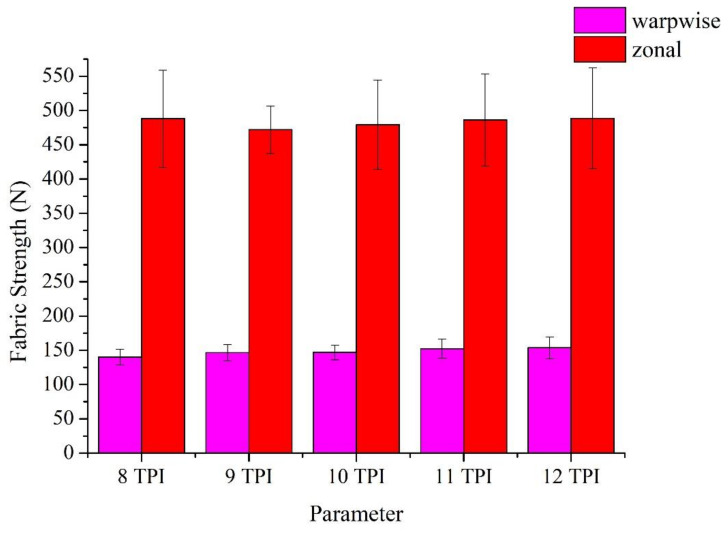
Tensile strength of non-woven fabrics composed of the Ge/SS core-spun yarns.

Figure 6 shows the tensile strength of woven fabrics that are composed of Ge/SS core spun yarns and 500D PET yarns. According to Table 2, the average tensile strength along the weft direction is between 140 N and 150 N The tensile strength along the weft direction is between 140 and 150 N, and the maximal tensile strength (153.6 N) of non-woven fabrics occurs when the constituent core-spun yarns are made of 12 TPI. The weft yarns are the core-spun yarns, and the tensile strength along the weft direction are in direct proportion to the TPI, indicating that the core-spun yarns are produced with an intact structure that is not jeopardized in the process. By contrast, the tensile strength along the warp direction does not fluctuate because the warp yarns are 500 D PET yarns exclusively.

**Figure 7:**
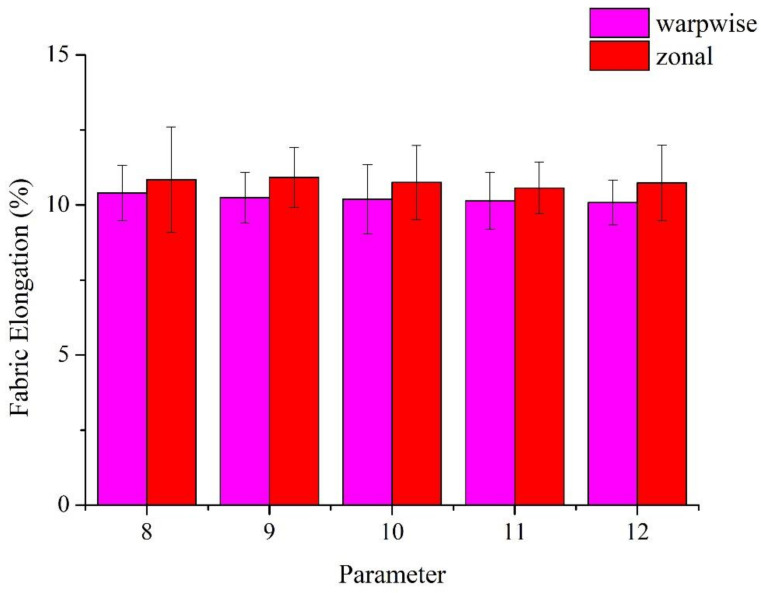
Tensile elongation of non-woven fabrics composed of the Ge/SS core-spun yarns.

Figure 7 and Table 3 shows the tensile elongation of the woven fabrics. It is the core-spun yarns that mainly distribute the exerted tensile force along the weft direction. The elongation along the weft direction shows a declining trend when the TPI increases. A higher TPI means that the stainless-steel filaments are twisted to a greater extent, which shows a negative influence over the elongation along the weft direction. By contrast, as the woven fabrics have the same warp yarns (i.e., 500D PET yarns), there is no significant difference in the elongation along the warp direction of woven fabrics.

### 3.3. Electromagnetic Wave Shielding of Woven Fabrics as Related to the TPI of Constituent Ge/SS Core-Spun Yarns

**Figure 8:**
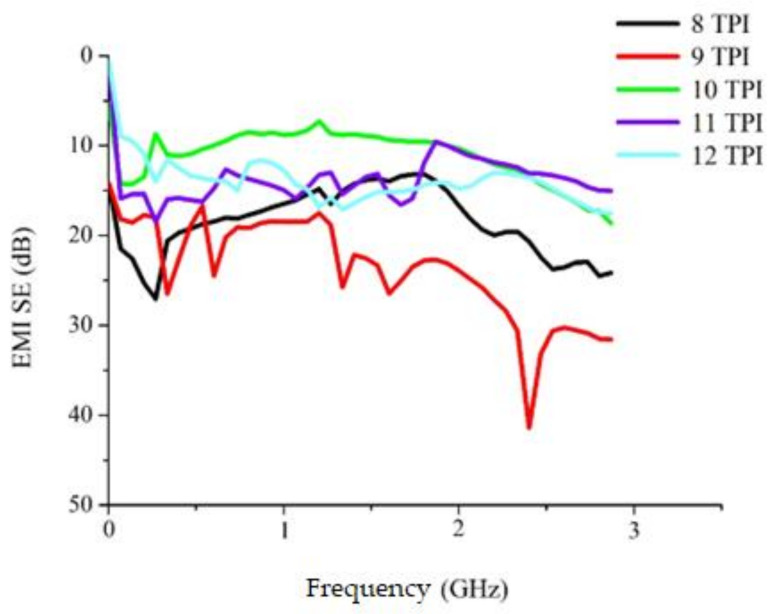
Electromagnetic wave shielding of non-woven fabrics composed of Ge/SS core-spun yarns.

A rise in the TPI gradually fails to wrap metallic core in Ge fibers evenly. When the TPI is 10, stainless steel wires begin to be exposed and cannot be in a linear status, the trend of which resembles the trend found in another study. A TPI exceeding 10 renders the non-woven fabrics with uneven voids among stainless steel filaments, which is less satisfactory than other woven fabrics made of a TPI lower than 10. Figure 8 shows that the optimal electromagnetic interference shielding effectiveness (EMI SE) occurs when the non-woven fabrics are composed of core-spun yarns of 8 or 9 TPI, outperforming higher TPI. The proposed woven fabrics demonstrate excellent EMI SE at low electromagnetic frequencies. Electromagnetic waves at different frequencies have different corresponding wave lengths, and the non-woven fabrics are made of a weft density that can dissipate electromagnetic waves via absorption loss and reflection loss. In summary, the non-woven fabrics of 9 TPI have a maximal EMI SE of −41.3 dB.

### 3.4. Air Permeability of Woven Fabrics as Related to TPI of Constituent Ge/SS Core-Spun Yarns

**Figure 9:**
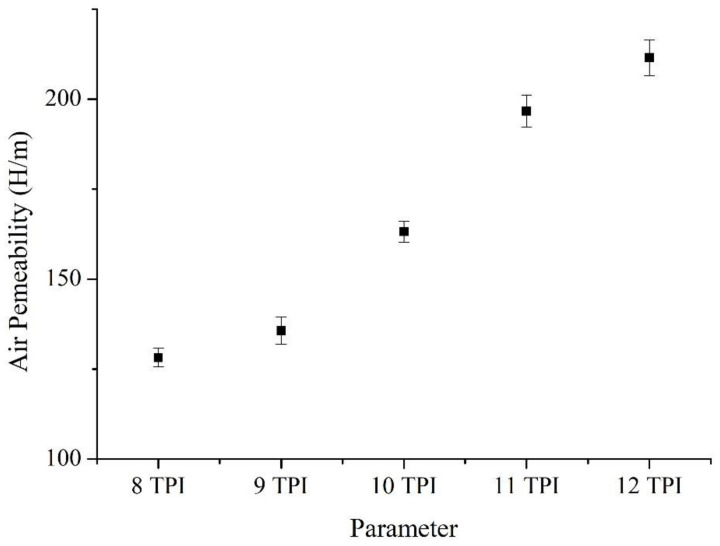
Air permeability of non-woven fabrics composed of Ge/SS core-spun yarns.

**Figure 10:**
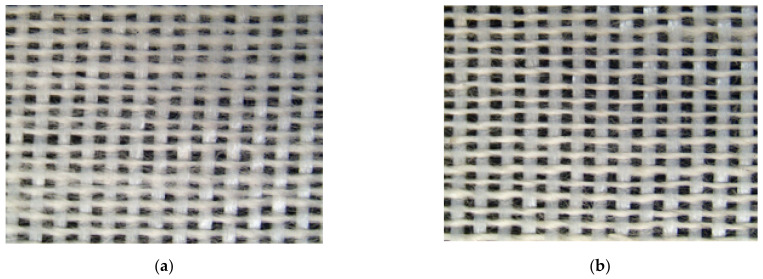
Surface image of non-woven fabrics composed of Ge/SS core-spun yarns with a TPI of (**a**) 8 and (**b**) 12.

Figure 9 and Table 4 shows that the air permeability of Ge/SS non-woven fabrics is in direct proportion to the TPI of the core-spun yarns. According to the surface observation of non-woven fabrics, Figure 10 shows that the core-spun yarns have a lower diameter and thus the non-woven fabrics have a more compact structure when TPI is increased. During the fabrication, the amount of hairiness in the woven fabrics is reduced, which in turn decreases the barriers against the air. Eventually the air permeability of non-woven fabrics is improved when the TPI increases.

### 3.5. Negative Ion Release of Woven Fabrics as Related to TPI of Constituent Ge/SS Core-Spun Yarns

**Figure 11:**
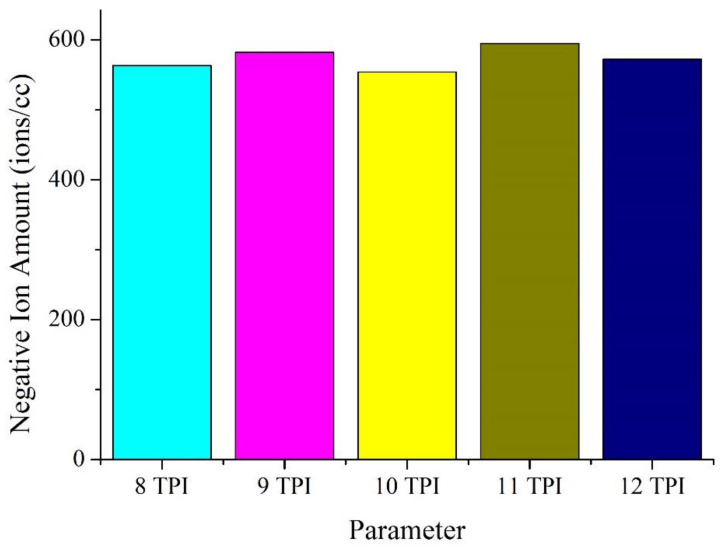
The release amount of negative ions of non-woven fabrics composed of the Ge/SS core-spun yarns.

Figure 11 and Table 5 shows that the woven fabrics composed of Ge/SS core-spun yarns can release negative ions, which is ascribed to the presence of Ge. Due to the identical materials of core-spun yarns, the negative ion amount does not differ by much when TPI changes. The non-woven fabrics release 500–600 ions/cc of negative ions, which is comparable to that of bamboo charcoal/stainless steel fabrics (350–400 ions/cc). Because of Ge/SS core-spun yarns, Ge/SS non-woven fabrics can release an optimal amount of negative ions that improve people’s metal stability, sleep quality, and appetite. A long-term contact with negative ions benefits individual health.

## 4. Conclusions

In this study, stainless steel filaments are wrapped in Ge fibers to form core-spun yarns, which is then combined with 500 D PET yarns for the production of woven fabrics. The fabrics exhibit electromagnetic wave shielding, negative ion release, and air permeability, and provide valuable applications. The core-spun yarns are made with 8–12 TPI using a ring frame and exhibit the maximal tensile strength of 5.0 N when made of 12 TPI. Based on the tensile strength and elongation of the yarns, it is surmised that the yarns may show an over-twisting phenomenon accompanied with a decreased tensile strength when the TPI exceeds 12. Made of the core-spun yarns with various TPI as well as 500 D PET yarns, woven fabrics demonstrate a maximal tensile strength of 153.6 N when the TPI is 12. Moreover, the air permeability of non-woven fabrics increases when TPI is increased. A higher TPI provides the yarns with a lower diameter, which is beneficial for the air permeability of the resultant non-woven fabrics. Specifically, when the constituent core-spun yarns are made of 8 or 9 TPI, the woven fabrics show excellent electromagnetic shielding when the electromagnetic frequency is lower than 1 GHz. In particular, a TPI of 9 provides the non-woven fabrics with the highest electromagnetic shielding of −41.3 dB. Finally, the proposed non-woven fabrics release a greater amount of negative ions (595 ions/cc) than the bamboo charcoal fiber fabrics (350 ions/cc).

## Figures and Tables

**Table 1:** Physical properties of Ge fiber/stainless steel core-spun yarns.

Yarn Twist (TPI)	Yarn Strength (N)	Yarn Elongation (%)	Yarn Diameter (mm)	Yarn Strength CV%	Yarn Tenacity (N/mm^2^)
8	3.0 ± 0.18	134.4 ± 11.2	0.327	6.0%	35.7 ± 2.1
9	3.8 ± 0.15	112.7 ± 7.79	0.304	4.0%	52.4 ± 2.0
10	4.3 ± 0.23	98.4 ± 17.04	0.288	5.4%	66.0 ± 3.5
11	4.5 ± 0.28	48.5 ± 5.27	0.275	6.2%	75.8 ± 4.7
12	5.0 ± 0.26	43.2 ± 3.51	0.245	5.3%	106.1 ± 5.5

**Table 2:** Tensile strength of non-woven fabrics as related to the TPI of constituent core-spun yarns.

Parameter	Yarn Strength (N)	Fabric Strength along the Warp Direction (N)	Fabric Strength along the Weft Direction (N)
8 TPI	3.0 ± 0.18	140.0 ± 11.5	488.1 ± 70.6
9 TPI	3.8 ± 0.15	146.6 ± 11.7	471.9 ± 34.4
10 TPI	4.3 ± 0.23	147.0 ± 10.8	479.0 ± 65.1
11 TPI	4.5 ± 0.28	152.4 ± 13.9	486.2 ± 66.9
12 TPI	5.0 ± 0.26	153.6 ± 15.9	488.7 ± 73.8

**Table 3:** Tensile elongation of non-woven fabrics composed of Ge/SS core-spun yarns.

Parameter	Yarn Elongation (%)	Fabric Elongation (%) (Warpwise)	Fabric Elongation (%) (Zonal)
8 TPI	3.0 ± 0.18	10.40 ± 0.92	10.84 ± 1.76
9 TPI	3.8 ± 0.15	10.25 ± 0.84	10.92 ± 1.00
10 TPI	4.3 ± 0.23	10.20 ± 1.15	10.75 ± 1.23
11 TPI	4.5 ± 0.28	10.14 ± 0.96	10.57 ± 0.86
12 TPI	5.0 ± 0.26	10.08 ± 0.75	10.74 ± 1.25

**Table 4:** Air permeability of non-woven fabrics.

PI	Air Permeability (H/m)
8	128
9	136
10	163
11	197
12	212

**Table 5:** Release amount of negative ions of non-woven fabrics.

TPI	Negative Ion Amount (Ions/cc)
8	563
9	582
10	554
11	595
12	572

## Data Availability

All data relevant to the study are included in the article.
